# Contribution of the precursors and interplay of the pathways in the phospholipid metabolism of the malaria parasite[S]

**DOI:** 10.1194/jlr.M085589

**Published:** 2018-05-31

**Authors:** Sharon Wein, Salma Ghezal, Corinne Buré, Marjorie Maynadier, Christian Périgaud, Henri J. Vial, Isabelle Lefebvre-Tournier, Kai Wengelnik, Rachel Cerdan

**Affiliations:** Dynamique des Interactions Membranaires Normales et Pathologiques, UMR 5235,* CNRS-Université de Montpellier, 34095 Montpellier Cedex 05, France; Institut des Biomolécules Max Mousseron, UMR 5247,† CNRS-Université de Montpellier, 34095 Montpellier Cedex 05, France; Chimie et Biologie des Membranes et des Nanoobjets, UMR 5248,§ Centre de Génomique Fonctionnelle, Université Bordeaux 2, 33076 Bordeaux Cedex, France

**Keywords:** *Plasmodium falciparum*, Kennedy pathway, phosphatidylcholine, phosphatidylethanolamine, phosphatidylserine, lysophosphatidylcholine, lipidomics, tandem mass spectrometry, stable isotope tracers

## Abstract

The malaria parasite, *Plasmodium falciparum*, develops and multiplies in the human erythrocyte. It needs to synthesize considerable amounts of phospholipids (PLs), principally phosphatidylcholine (PC), phosphatidylethanolamine (PE), and phosphatidylserine (PS). Several metabolic pathways coexist for their de novo biosynthesis, involving a dozen enzymes. Given the importance of these PLs for the survival of the parasite, we sought to determine their sources and to understand the connections and dependencies between the multiple pathways. We used three deuterated precursors (choline-d_9_, ethanolamine-d_4_, and serine-d_3_) to follow and quantify simultaneously their incorporations in the intermediate metabolites and the final PLs by LC/MS/MS. We show that PC is mainly derived from choline, itself provided by lysophosphatidylcholine contained in the serum. In the absence of choline, the parasite is able to use both other precursors, ethanolamine and serine. PE is almost equally synthesized from ethanolamine and serine, with both precursors being able to compensate for each other. Serine incorporated in PS is mainly derived from the degradation of host cell hemoglobin by the parasite. *P. falciparum* thus shows an unexpected adaptability of its PL synthesis pathways in response to different disturbances. These data provide new information by mapping the importance of the PL metabolic pathways of the malaria parasite and could be used to design future therapeutic approaches.

Malaria is a major global health problem, affecting more than 40% of the world’s population. This disease is responsible for 196 million to 263 million annual cases, resulting in about 445,000 deaths, most of them occurring in children under the age of 5 in sub-Saharan Africa (1). Malaria is caused by parasites of the genus *Plasmodium*, and the most lethal human malaria parasite is *Plasmodium falciparum*. The parasite has a complex life cycle developing in mosquitos and humans, but all clinical features are caused during the repeated invasion of human red blood cells (RBCs). No efficient vaccine is currently available, and resistance to all available treatments has now been observed, making it urgent to identify novel pharmacological targets (1–3). The mature RBC is a very particular host cell for an intracellular parasite. RBCs do not contain organelles and are therefore capable of only few metabolic functions. After invasion, the parasite resides and develops within a parasitophorous vacuole and produces an extensive tubular membrane network in the erythrocyte cytoplasm. During its development, the parasite internalizes and digests the host cell hemoglobin (Hb) in a particular proteolytic compartment called the food vacuole (FV) (4). All other nutrients and metabolic precursors that are essential for parasite survival must either be imported from the serum or be synthesized de novo by the parasite.

During the intraerythrocytic phase, *P. falciparum* synthesizes considerable amounts of membranes for its growth within the host cell and for the subsequent formation of up to 32 daughter cells every 48 h. This is associated with a 6-fold increase in the phospholipid (PL) content of the erythrocyte infected with the metabolically highly active late stages of the parasite (trophozoite and schizont) (5, 6). Indeed, *Plasmodium* membranes are mainly composed of PLs and contain only little cholesterol (7, 8). In *P. falciparum*-infected RBCs (iRBCs), PLs constitute 60% of the total lipid content versus 38% in uninfected RBCs (uRBCs) (5). In uRBCs, the main PLs are phosphatidylcholine (PC) (30–40%), phosphatidylethanolamine (PE) (25–35%), SM (15%), and phosphatidylserine (PS) (10–20%) (9, 10). After infection by *P. falciparum*, an increase of PC and PE and a decrease of PS and SM are observed. In *P. falciparum*-iRBCs as well as in purified parasites, the main PLs are PC (45–55%), PE (20–35%), PS (4–8%), phosphatidylinositol (∼5%), and SM (3–20%) (5, 11, 12). De novo synthesis of lipids is absent in uRBCs (10), but *Plasmodium* possesses its own machinery to produce PLs (7, 13). Previous studies have shown that PL synthesis is essential for the viability of *P. falciparum* at the blood stage (14, 15). Potent antimalarial activity has been observed with choline (Cho) analogs that target PC synthesis. The lead compound, albitiazolium, was successfully evaluated during human phase I and II clinical trials in adult patients. However, phase II clinical trials in a pediatric population revealed a lower efficiency due to higher drug clearance as compared with adults and led to an arrest of the clinical development (15, 16). Targeting PC synthesis in *P. falciparum* is thus a proven strategy. It is therefore even more important to decipher the PL metabolism in order to identify additional pharmacological targets.

The PL metabolism of *P. falciparum* is unique in its intensity and in the multiplicity of its pathways. Its metabolism combines pathways generally found in prokaryotes and eukaryotes. The three main PLs are PC, PE, and PS. PC can be synthesized from Cho via the three enzymatic steps of the CDP-Cho-dependent Kennedy pathway (17–19) (**Fig. 1**). However, PC can also be synthesized from ethanolamine (Etn) that enters the same pathway after the triple methylation of phosphoethanolamine (P-Etn) to phosphocholine (P-Cho) by the P-Etn methyltransferase (PMT), an enzyme generally found in plants (20). On the other hand, triple methylation of PE to PC, as present in mammalian cells, is absent in *P. falciparum* (21). PE can be synthesized via the CDP-Etn-dependent Kennedy pathway from Etn (17, 19). For PC and PE synthesis, Etn can also be provided by the decarboxylation of serine (Ser) by the Ser decarboxylase (22), an enzyme generally found in plants (23). The activity of this enzyme has been detected in the parasite (22), but its corresponding gene has still not been identified. PE can also be generated by decarboxylation of PS through the PS decarboxylase (PSD) (24). Finally, biosynthesis of PS from Ser has been demonstrated biochemically in *P. falciparum* (25). The only PS synthase (PSS) present in *P. falciparum* (PF3D7_1366800) is similar to the mammalian PS synthase 2 (PSS2), which catalyzes the base-exchange reaction between PE and Ser (Fig. 1). A previously predicted CDP-diacylglycerol (DAG)-dependent PSS enzyme (7) has now been reannotated as a phosphatidylglycerolphosphate synthase (PF3D7_0820200) in the *Plasmodium* database (www.plasmodb.org).

**Fig. 1.**
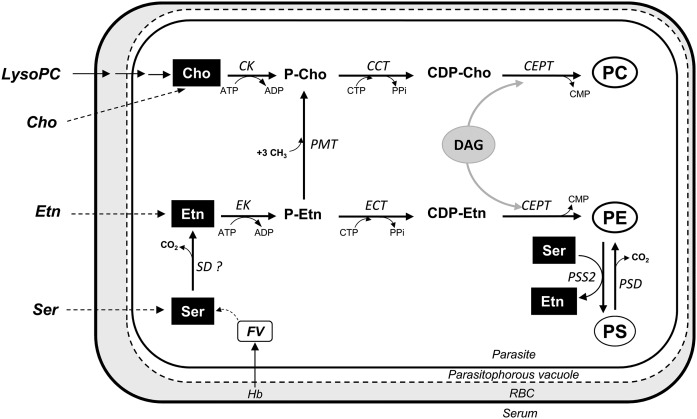
Biosynthesis pathways of the three main PLs of *P. falciparum* (PC, PE, and PS) at the intraerythrocytic stage. Dashed arrows correspond to nonenzymatic steps. The “?” indicates that the enzyme activity was reported but the gene is not yet identified. CK, choline kinase; CCT, CTP:phosphocholine cytidylyltransferase; CEPT, choline/Etn-phosphotransferase; ECT, CTP:phosphoethanolaminecytidylyltransferase; EK, Etn kinase; PPi, pyrophosphate; SD, serine decarboxylase.

In the present study, we determined the contribution of the different pathways to the biosynthesis of PC, PE, and PS, and we analyzed their regulation. To this purpose, we undertook a lipidomic analysis of *P. falciparum*-iRBCs using deuterium-labeled Cho, Etn, and Ser as tracers to follow the different PL pathways (supplemental Fig. S1). We identified the reaction intermediates and the final PL products of each pathway by LC/MS/MS and quantified the relative contribution of each pathway to the biosynthesis of the three main PLs. When we started our study, Cho from serum (10–15 µM) (Human Metabolome Data Base, http://www.hmdb.ca) was considered to be the only source of Cho for the parasite. Our results suggest that the parasite uses almost exclusively lysophosphatidylcholine (LysoPC) from serum to synthesize PC through the Kennedy pathway. In accordance with our data, Brancucci et al. showed recently that the majority of free Cho in the parasite derives from LysoPC (26). PE can be provided by both Etn and Ser by three different routes. We also show that Ser used for PS synthesis is predominantly provided by Hb degradation. Comparative lipidomics with a transgenic parasite line in which the gene encoding PMT activity is deleted clarified the interplay between the different pathways showing that *P. falciparum* is able to compensate for the lack of Cho by using Etn or Ser.

## METHODS

### Chemicals

Metabolites LysoPC and SM from egg yolk and internal standards were obtained from Sigma-Aldrich, with the exception of cytidine-5′-diphosphate, which was purchased from MP Biomedicals Inc. (Illkirch, France) and myo-inositol-1-phosphate from Interchim (Montluc, France). LysoPC contains primarily palmitic and stearic acids. SM contains primarily palmitic acid. All the deuterated compounds were purchased from CDN Isotopes (Québec, Canada). RPMI 1640 medium and RPMI 1640 without Cho, Ser, methionine, inositol, and folic acid were obtained from Gibco.

### Biological material

*P. falciparum* (3D7 and *pfpmtΔ* strains) cell cultures were performed as previously described (27) in human A^+^ erythrocytes and complete medium (RPMI 1640 medium supplemented with 25 mM HEPES) and 10% AB^+^ human serum or 0.5% Albumax I (Gibco), at 37°C under a gaseous mixture of 5% CO_2_, 5% O_2_, and 90% N_2_. Parasites were synchronized using a VarioMACS magnetic cell separator (CS column, Miltenyi Biotec, Paris, France) followed by a 5% sorbitol treatment (28, 29). Free parasites were prepared from mature infected erythrocytes by adding 10 vols of 0.02% (wt/vol) saponin in cold PBS (116 mM NaCl, 8.3 mM Na_2_HPO_4_, and 3.2 mM KH_2_PO_4_, pH 7.4) for 1 min, followed by two washes by centrifugation at 1,260 *g* for 5 min at 4°C and resuspension in PBS.

Uninfected human blood and serum were provided by the local blood bank (Etablissement Français du Sang) under the approval number 21PLER2016-0103.

### Metabolic labeling

For metabolic labeling, RBCs and iRBCs at late stages were first washed three times in modified RPMI 1640 and put back into culture in the appropriate medium. Culture medium was composed of modified RPMI 1640 supplemented with 20 mM HEPES buffer (Gibco, pH 7.4), glutamine (0.3 g/l), hypoxanthine (15 µg/ml), inositol (0.035 g/l), gentamycin (40 µg/ml), deuterated precursors (4–1,000 µM Cho-d_9_, 2–500 µM Etn-d_4_, and 20–1,000 µM Ser-d_3_), and 10% human serum AB^+^ or 0.5% Albumax I. Serum-free culture medium was prepared according to Mitamura et al. (30) as follows. Lipid-free BSA stock solution [600 µM in RPMI 1640 without Cho, Ser, and inositol (Gibco) (modified RPMI)] was added to dried lipid precipitate (oleic acid and palmitic acid, 30 µM final), and the mixture was sonicated for 1 min to allow FAs to bind to BSA. The final concentration of lipid-free BSA was 30 µM. This solution was supplemented with 20 mM HEPES buffer (Gibco, pH 7.4), glutamine (0.3 g/l), hypoxanthine (15 µg/ml), inositol (0.035 g/l), 140 µM Ser, 10 µM Etn, and gentamycin (40 µg/ml). Then, serum-free culture medium was supplemented or not with 20 or 400 µM Cho, or 20 µM LysoPC or 20 µM SM. For supplementation with 20 µM LysoPC or SM, lipid-free BSA stock solution was added to dried lipid precipitate containing oleic acid, palmitic acid, and LysoPC or SM and then sonicated. FAs, LysoPC, and SM were thus bound to BSA, mimicking serum conditions and avoiding micelle formation. In this case, the final concentration of lipid-free BSA was 40 µM. When labeling was done in serum-free culture medium, Etn and Ser were replaced by 10 µM Etn-d_4_ and 140 µM Ser-d_3_ and supplemented with 20 µM Cho-d_9_.

For labeling experiments, the parasite cultures were washed in modified RPMI and then maintained in culture medium containing deuterated precursors for 48 or 96 h at 37°C, under an atmosphere of 5% CO_2_, 5% O_2_, and 90% N_2_. Medium was changed every 24 h. Lipidomic analyses were generally performed on iRBCs (and not on isolated parasites) because these preparations contained all membrane compartments, within the parasite and exported by the parasite to the host erythrocyte. For this, mature iRBCs were purified using VarioMACS. Occasionally, parasites were isolated from mature iRBCs using 0.01% saponin for 1 min at 4°C in cold PBS buffer. Cells were washed with cold PBS buffer, resuspended in 100 µl of water, and stored at −80°C until extraction of water-soluble metabolites. Pellets for extraction of the PLs were directly stored at −80°C.

### Extraction of reaction intermediates and PLs

Water-soluble metabolites were extracted according to the method of Storm et al. (31). Briefly, after rapid thawing, 400 µl of cold chloroform/methanol (1/3, vol/vol) was added to cell lysate or culture medium aliquot. Samples were then vigorously mixed, sonicated at 4°C using a sonicating water bath, and incubated at 4°C under shaking conditions during 1 h. After centrifugation at 16,000 *g* at 4°C, the supernatant was stored at −80°C until LC/MS/MS analysis. The cellular lipids were extracted according to the procedure of Folch et al. (32), as modified by Rock (33). Briefly, after rapid thawing, 2.1 ml of methanol was added to 350 µl of cell pellet and shaken vigorously. After 15 min at 4°C, 1.05 ml of chloroform was added, and the sample was incubated during 30 min at room temperature, then centrifuged 10 min at 450 *g*. The supernatant was collected, and the pellet was resuspended in 1.68 ml of chloroform/methanol/water (37.5/50/12.5: vol/vol/vol). After 10 min of centrifugation at 1,800 *g*, the supernatant was collected and pooled, and then 4.2 ml of chloroform and 1.75 ml of water were added. After 10 min under shaking conditions, the sample was centrifuged 10 min at 1,800 *g*, and the aqueous phase was removed. The organic phase was dried with nitrogen and resuspended in 100 µl of chloroform/methanol (2:1), transferred into vials under nitrogen, and stored at −80°C until LC/MS/MS analysis.

### Characterization and relative quantification of PLs by reverse-phase LC/MS/MS

Reverse-phase LC (RPLC) was carried out at 40°C on a Luna 5u C8 (2) 100 Å, 150 × 1 mm, 5 µm particles (Phenomenex, Le Pecq, France). The gradient elution program was a combination of eluent A (isopropanol/methanol/water: 50/10/40: vol/vol/vol) with 0.2% formic acid and 0.028% ammonium hydroxide and eluent B (isopropanol with 0.2% formic acid and 0.028% ammonium hydroxide) with 30% B (0 min), 50% B (0–5 min), 80% B (5–30 min), and 30% B (30–35 min). The flow rate was set at 40 µl × min^−1^, and 3 µl sample volumes were injected. RPLC/MS/MS analyses were performed with a 5500 QTRAP (AB Sciex) instrument coupled to an LC system (Ultimate 3000, Dionex). Five microliters of PL extracts dissolved in isopropanol/methanol/water (50/10/40: vol/vol/vol) (3.75 × 10^5^ cells/µl) was added to 5 µl of internal standards (PE and PC at 5 µM and PS at 10 µM) and dissolved in 40 µl of isopropanol/methanol/water (50/10/40: vol/vol/vol). Analyses were carried out in the negative (PE and PS) and positive (PC) modes with fast polarity switching (50 ms). RPLC/MS/MS method in multiple reaction monitoring (MRM) mode was adapted for MRM transitions from Buré et al. (34). Nitrogen was used as curtain gas (set to 20), gas1 (set to 25), and gas2 (set to 0). Needle voltage was at −4,500 or + 5,500 V without needle heating; declustering potential was between −180 and −85 V or set at +40 V. The collision gas was nitrogen; collision energy varied from 47 to 62 eV on a compound-dependent basis. The dwell time was set to 3 ms. MS/MS experiments were performed by 73 positive MRM scans and 300 negative MRM scans according to the PL identifications obtained by shotgun MS. The area of LC peaks was determined using MultiQuant software (version 3.0, AB Sciex). Only labeled and unlabeled PC, PE, and PS were searched for and quantified in the samples.

### Characterization of PL species by shotgun-mass spectrometry

Shotgun-mass spectrometry analysis by ESI was carried out according to the protocol previously described (34). Dried PL extracts were dissolved in 500 µl of a mixture of chloroform/methanol (2/1: vol/vol) containing 7.5 mM ammonium acetate. Each sample was infused into the TurboV electrospray source of a QTRAP 5500 mass spectrometer (AB Sciex, Concord, Canada) at a flow rate of 7 µl·min^−1^, with fast polarity switching (50 ms) at a scan rate of 200 Da/s. ESI-MS/MS experiments in precursor ion scan were performed in negative ion mode to screen for PE and PS and led to 53 precursor ion scans. In positive ion mode, one precursor ion scan allowed screening for PC. PL species were identified using Lipid View software (version 1.2, AB Sciex).

### Reaction intermediates analysis by LC/MS/MS

The analysis of selected reaction intermediates was performed by the method of Vo Duy et al. (35). LC instrumentation included an ACQUITY^TM^ Ultra Performance Liquid Chromatography integrated system from Waters (Milford, MA). Mass spectrometry experiments were performed using a triple quadrupole mass spectrometer TSQ Quantum Ultra^TM^ (Thermo Fisher Scientific Inc., Waltham, MA). Metabolites were separated using an Acquity UPLC BEH Shield RP_18_ column (150 mm × 2.1 mm, 1.7 µm; Waters, Saint-Quentin en Yvelines, France) in the positive ionization mode. Heptafluorobutyric acid was added to the mobile phase as an ion pair reagent. Quantitation was performed using one internal standard. Each metabolite was quantified against a calibration curve prepared by spiking RBC cell pellets (3 × 10^6^ cells) with appropriate working solutions of all metabolites and the internal standard. Using the method Vo Duy et al. (35) the quantities of P-Cho were overestimated because of a lack of specificity. The introduction of a second product ion for P-Cho allowed us to increase the specificity of the method and to obtain correct values.

For the water-soluble metabolites and the final PLs, the area of peaks were determined and reported as percent of the total contents of the corresponding metabolite or PL.

### Calculation of Etn and Ser contributions to PE biosynthesis

PE can be provided by the Kennedy pathway or by the PSD pathway. To distinguish between the two origins, we assume that the relative amounts of CDP-Etn are conserved in the relative amounts of PE. In other words, the 56.8% of CDP-Etn from Etn-d_4_ gave the 34.7% of PE from Etn-d_4_. Conserving this ratio, we calculated that 6.4% of CDP-Etn from Ser-d_3_ should correspond to 3.9% of PE from Ser-d_3_, and 36.8% of CDP-Etn from unlabeled Ser should give 22.5% of unlabeled PE. The remaining PE corresponds to the contribution of the PSD pathway and to the PE of the erythrocyte membrane. To summarize, the Kennedy pathway contributes to 61.1% of PE biosynthesis (34.7% of PE from Etn-d_4_, 22.5% of PE from unlabeled Ser, and 3.9% of PE from Ser-d_3_). The PSD pathway provides 18.9% of PE (14.3% of PE from unlabeled Ser and 4.6% of PE from Ser-d_3_). The remaining 20% of PE originates from unlabeled PE of the erythrocyte membrane.

### Parasite growth assays

uRBCs and *P. falciparum*-iRBCs were washed three times in modified RPMI 1640 and put back into six different culture media during 5 days. Culture media were RPMI 1640 supplemented with 10% human serum (control) or serum-free medium supplemented or not with 20 µM Cho, 400 µM Cho, 20 µM LysoPC, or 20 µM SM. Giemsa smears of the cultures were done daily, and parasitemia were determined by light microscopy.

## RESULTS

### Contributions of the different precursors to the PL biosynthesis pathways

**Fig. 2.**
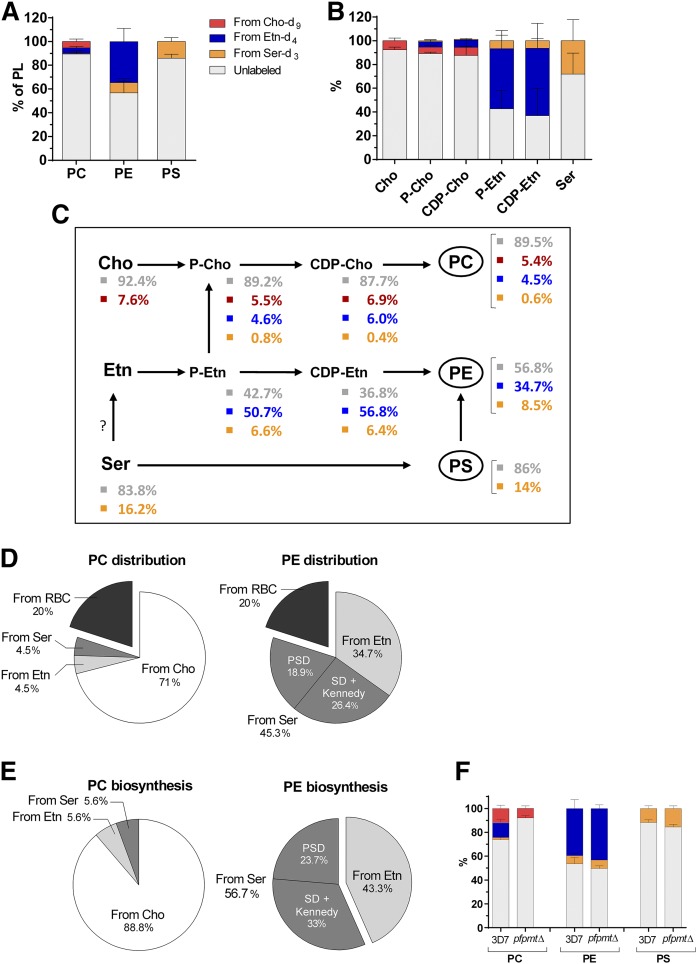
Contributions of Cho, Etn, and Ser to *P. falciparum* PL metabolism. *P. falciparum*-iRBCs (3D7 and *pfpmtΔ* strains) were incubated 48 h in culture medium supplemented with 10% human serum in the presence of 20 µM Cho-d_9_, 10 µM Etn-d_4_, and 140 µM Ser-d_3_. After incubation, PLs and reaction intermediates were quantified by LC-MS/MS. The relative contributions of Cho-d_9_, Etn-d_4_, and Ser-d_3_ in the 3D7 strain to biosynthesis of PLs (A) and reaction intermediates (B) are presented. The color code used here is conserved for all figures. Results are shown as means ± SD (n≥3). C: Summary diagram of PL biosynthesis pathways of 3D7 strain showing the sources of PL and reaction intermediates. Values with SD are given in supplemental Table 1. D, E: Relative precursor contributions to PC and PE (D) and biosynthesis (E). For PE, distinction was made between PE coming from Ser decarboxylation and the CDP-Etn-dependent pathway (Ser decarboxylase + Kennedy) and PE coming from PS decarboxylation (PSD). PS was synthesized by a single biosynthesis pathway from Ser and is not represented here. F: Contributions of Cho-d_9_, Etn-d_4_, and Ser-d_3_ to biosynthesis of PL in the *pfpmtΔ* strain. Results are shown as means ± SD (*n* = 3).

To determine the contributions of the different pathways to PC, PE, and PS biosynthesis, RBCs infected with the *P. falciparum* 3D7 strain were incubated in a particular culture medium containing physiologically relevant concentrations of the three deuterium-labeled PL precursors Cho-d_9_, Etn-d_4_, and Ser-d_3_ (supplemental Fig. S1). Unlabeled precursors were absent from this culture medium, with the exception of low concentrations of Cho and Ser originating from human serum. In order to maximize metabolic labeling, we chose to incubate synchronized parasites at the trophozoite stage during 48 h to allow for a full developmental cycle, including a reinvasion step. PLs and reaction intermediates were then quantified by LC/MS/MS (34–36). Surprisingly, a large majority of PLs synthesized during 48 h in the continuous presence of deuterated precursors in the culture medium were found to be unlabeled. We determined that 89.5 ± 3.0% of PC, 56.8 ± 12.0% of PE, and 86 ± 3% of PS was unlabeled (**Fig. 2A** and supplemental Table S1). This was also the case for the PL precursors and reaction intermediates (Fig. 2B and supplemental Table S1). As human serum contains unlabeled precursors, we repeated the experiments using Albumax, a Cho- and Ser-free serum substitute. Again, similar high levels of unlabeled PLs were detected, suggesting that the unlabeled PLs did not derive from Cho or Ser present in the culture medium (supplemental Fig. S2A). The results also showed that the level of unlabeled precursors Cho and Ser was: *i*) much higher in iRBCs as compared with uRBCs in both culture conditions and *ii*) comparable in both culture conditions (supplemental Fig. S2B, C). Finally, unlabeled PLs were also predominant in isolated parasites, indicating that their presence was mainly imputed to the parasite itself (supplemental Fig. S2D). A certain fraction of unlabeled PLs originates from the erythrocyte membrane that remains unlabeled due to the absence of PL biosynthesis in these cells. It has previously been shown that the total PL content of an iRBC at the trophozoite stage is about 6 times higher than in an uRBC (6). It can thus be deduced that about one-sixth of the total PL content corresponds to the host erythrocyte membrane (from 15% to 20%). In the following interpretations of the results, we assigned 20% of the unlabeled PLs to the PLs of the erythrocyte membrane. Fig. 2C summarizes the distribution of the three labeled precursors at each step of the biosynthesis of PC, PE, and PS. Cho was mainly found unlabeled (92.4%) and likely derived from LysoPC of the culture medium (26). Ser was also predominantly found unlabeled (83.8%). The current annotation of the *Plasmodium* genome suggests that Ser cannot be synthesized by the parasite. The sources of Ser can therefore only be the serum (or culture medium in vitro) and the amino acids released by the massive Hb degradation in the parasite FV (4). Our analysis showed that the serum used in our experiments was Etn-free (supplemental Fig. S2C). Etn can thus be provided by exogenous Etn-d_4_, exogenous Ser-d_3_, or unlabeled Ser (Fig. 2C). Unfortunately, the quantification of Etn in iRBCs was not possible due to Etn leakage out of the cells during the washing steps. However, it is likely that the Etn distribution was similar to the one of P-Etn. In this case, we can suppose that half of the Etn was labeled as Etn-d_4_ and the other half was provided by Ser decarboxylation (d_3_-labeled and unlabeled). We observed that the contributions of each precursor (labeled and unlabeled) were comparable for both intermediate metabolites of the Kennedy pathways (P-Cho/CDP-Cho and P-Etn/CDP-Etn).

In order to evaluate the contribution of each precursor to each pathway, we first analyzed the results obtained for the PLs. Concerning PS biosynthesis, 14% was synthesized from Ser-d_3_; the 86% of unlabeled PS could be attributed to 20% from erythrocyte membrane and 66% from Ser provided by Hb degradation (Fig. 2C). The interpretation of the results for PC was less straightforward because the three precursors contributed to PC biosynthesis, following different pathways. To calculate their relative contributions to the total amount of unlabeled PC, we used the ratios found for P-Etn that indicated that about half was derived from Etn (Etn-d_4_) and the other half from Ser (d_3_-labeled and unlabeled). The 4.5% of PC derived from Etn-d_4_ thus indicated a similar contribution derived from Ser and, in this case, 0.6% from Ser-d_3_ and ∼3.9% from unlabeled Ser. Accordingly, the contributions to the PC pool were assigned as follow: 4.5% from Etn, 4.5% from Ser (0.6% from Ser-d_3_ and 3.9% from unlabeled Ser), 71% from Cho (5.4% from Cho-d_9_ and 65.6% from exogenous unlabeled LysoPC), and 20% from the erythrocyte membrane (unlabeled PC) (Fig. 2D). For PE biosynthesis, 34.7% was provided by Etn, 45.3% came from Ser (8.5% Ser-d_3_ and 36.8% unlabeled Ser), and 20% was from the erythrocyte membrane (Fig. 2D). However, two pathways were possible to synthesize the 45.3% of PE from Ser. Based on the ratios found for CDP-Etn, we estimated that 26% of PE was synthesized via Ser decarboxylation and the Kennedy pathway and 19% via PS decarboxylation (see Methods) (Fig. 2D). To better visualize the relative contributions of each precursor to the biosynthesis of the PLs, we recalculated our data excluding the 20% contribution of unlabeled PL of the host cell membrane (Fig. 2E). PC was predominantly synthesized from Cho (88.8%). PE was almost equally synthesized from Ser (56.7%) and Etn (43.3%). Among the two pathways coming from Ser, Ser decarboxylation was slightly more dominant (33%). All PS was derived from Ser through a unique route.

To assess the importance of the transversal pathway in PC biosynthesis, i.e., the trimethylation of P-Etn, we performed the labeling experiments on parasites lacking the *Pf*PMT (Fig. 1). In these *pfpmt* KO parasites (*pfpmtΔ*), we did not observe any PC biosynthesis from Etn-d_4_ or Ser-d_3_ (Fig. 2F). The proportion of PC synthesized from Cho-d_9_ was similar to the 3D7 strain (11.8 ± 2.6% vs. 8.0 ± 2.2%), thereby also confirming that there is no PE methyl transferase activity independent of *Pf*PMT (21). No changes were observed for PE and PS biosynthesis between the WT (3D7) and the *pfpmtΔ* strain (Fig. 2F).

### Effects of varying precursor concentrations on PL biosynthesis pathways

**Fig. 3.**
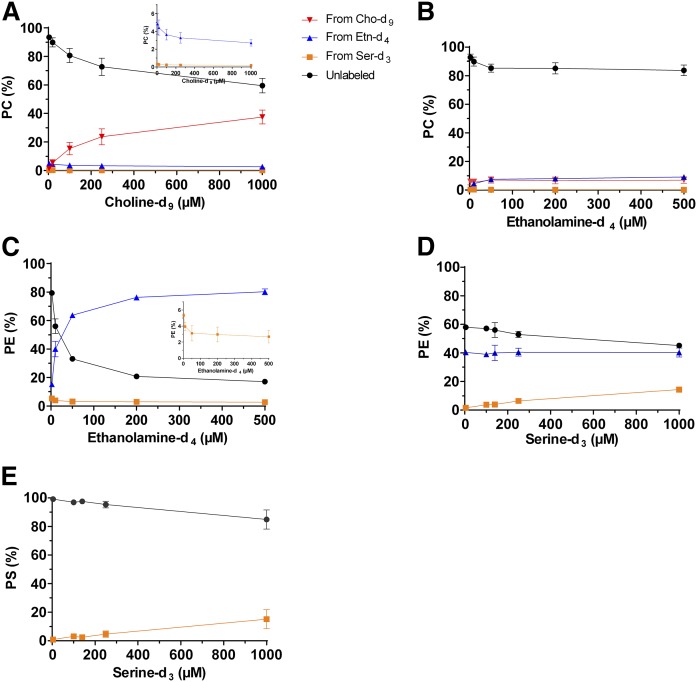
Effect of precursor concentrations on the PC, PE, and PS biosynthesis pathways in *P. falciparum*-iRBCs. *P. falciparum*-iRBCs were incubated 96 h in culture medium supplemented with 10% human serum in the presence of increasing concentrations of Cho-d_9_ (A), Etn-d_4_ (B, C), or Ser-d_3_ (D, E), while the two others labeled precursors remained at fixed concentrations of 20 µM Cho-d_9_, 10 µM Etn-d_4_, and 140 µM Ser-d_3_. After incubation, PLs were quantified by LC/MS/MS. Results are shown as means ± SD (*n* = 3). The same color code is used for all panels. The insets in A and C represent an enlargement of the graph for PC and PE values from Etn-d_4_ and/or Ser-d_3_.

To study the regulation of the multiple PL biosynthesis pathways, we determined the levels of labeled and unlabeled PLs upon increasing concentrations of one labeled precursor. *P. falciparum*-iRBCs were incubated during 96 h (i.e., two developmental cycles) in the presence of increasing concentrations of Cho-d_9_, Etn-d_4_, or Ser-d_3_, while the two other labeled precursors were kept at fixed concentrations. Increasing the Cho-d_9_ concentration led to higher levels of PC from Cho-d_9_, with a concomitant decrease of unlabeled PC and of PC from Etn-d_4_ (**Fig. 3A**). No significant variation of PC from Ser-d_3_ was observed. Even at the highest concentration of 1 mM Cho-d_9_, the fraction of unlabeled PC was still 60% of total PC (Fig. 3A). Free Cho-d_9_ in the culture medium was thus able, to some extent, to replace LysoPC as a Cho source. When the concentration of Etn-d_4_ was increased, a slight but significant decrease of unlabeled PC with an increase of PC from Etn-d_4_ was also observed (Fig. 3B). Increasing the Ser concentrations did not affect the balance of the PC biosynthesis pathways (supplemental Fig. S3A). With increasing Etn-d_4_ concentrations in the culture medium, unlabeled PE was efficiently replaced by PE from Etn-d_4_ (Fig. 3C), and also PE from Ser-d_3_ decreased. At 500 µM Etn-d_4_, only 20% of unlabeled PE remained, probably corresponding to the PE of the erythrocyte membrane. High extracellular Etn concentrations compelled the parasite to use Etn instead of Ser to synthesize PE. Increasing Ser-d_3_ concentrations induced only a slight increase of PE from Ser-d_3_ and a slight decrease of unlabeled PE, which reflected the replacement of unlabeled Ser by Ser-d_3_ (Fig. 3D). High Ser-d_3_ concentrations also had little impact on PS labeling, as revealed by a <20% decrease of the amount of unlabeled PS (Fig. 3E). This could probably be due to the continuous supply of unlabeled Ser coming from Hb degradation in the FV. No modification of PS labeling was observed when the concentrations of Cho-d_9_ and Etn-d_4_ were increased (supplemental Fig. S3C, D).

### Impact of LysoPC depletion on PC biosynthesis

**Fig. 4.**
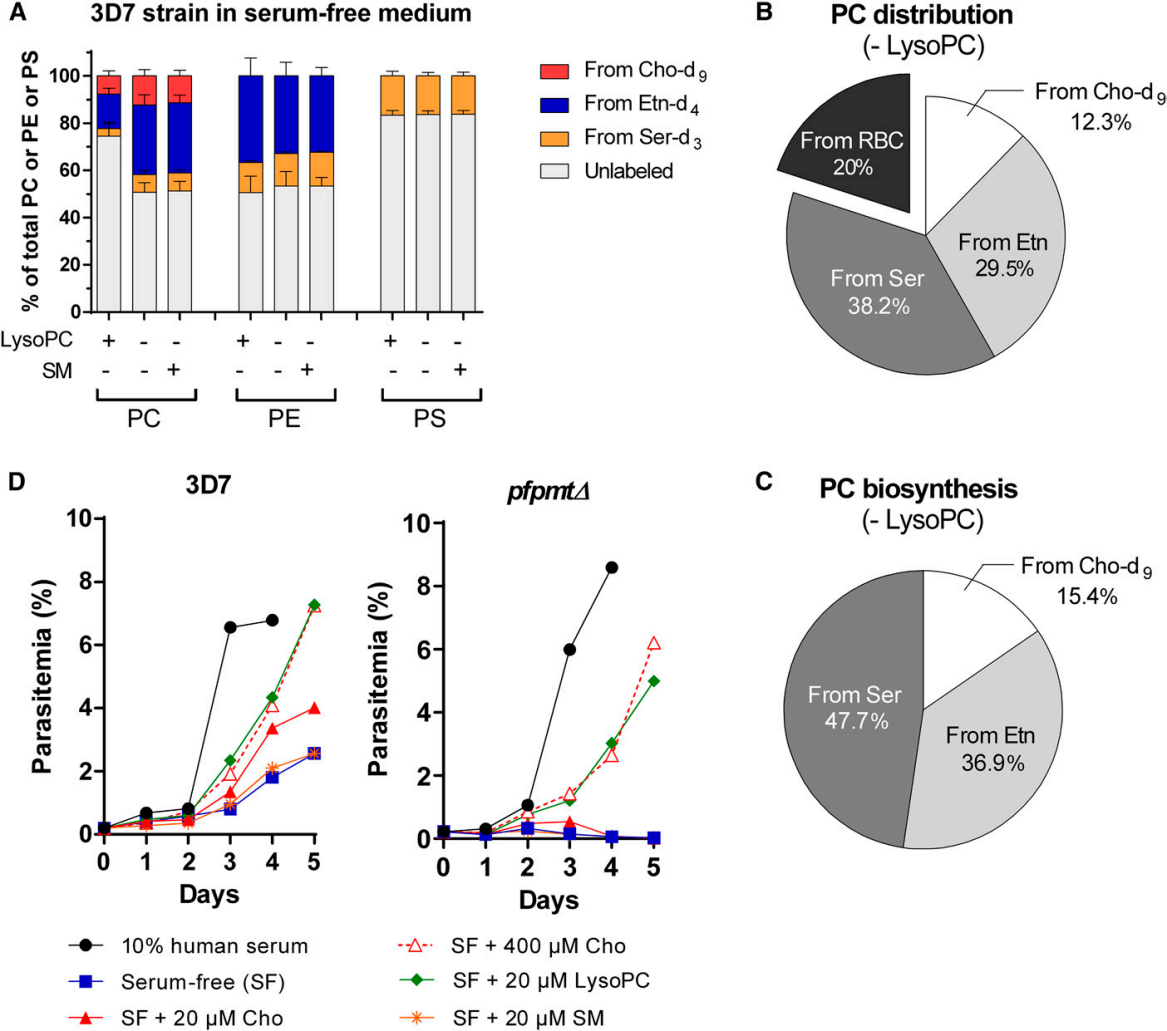
Influence of PMT and LysoPC on PC biosynthesis in *P. falciparum.* A: Contributions of Cho-d_9_, Etn-d_4_, and Ser-d_3_ to biosynthesis of PL in the *P. falciparum* 3D7 strain. *P. falciparum*-iRBCs were incubated for 48 h in serum-free medium with 20 µM Cho-d_9_, 10 µM Etn-d_4_, and 140 µM Ser-d_3_ and supplemented or not with 20 µM LysoPC or 20 µM SM. After incubation, PLs were quantified by LC/MS/MS. Results are shown as means ± SD (*n* = 3). B, C: Relative precursors contribution to PC content (B) and biosynthesis (C) in the absence of LysoPC. *P. falciparum*-iRBCs were incubated for 48 h in serum-free medium with 20 µM Cho-d_9_, 10 µM Etn-d_4_, and 140 µM Ser-d_3_. Results are shown as means ± SD (*n* = 3). D: Effect of LysoPC on growth of 3D7 and *pfpmtΔ* strains. *P. falciparum*-iRBCs were cultivated in culture medium supplemented with 10% human serum (control) or in serum-free medium supplemented or not with 20 µM Cho, 400 µM Cho, 20 µM LysoPC, or 20 µM SM. One representative experiment (of at least three experiments) is shown.

LysoPC has recently been shown to be the main source of Cho (26) and consequently of PC. We therefore analyzed the impact of LysoPC depletion on PC biosynthesis. We performed the labeling experiments with the *P. falciparum* 3D7 strain in a different culture medium without serum, supplemented or not with 20 µM LysoPC. In comparison to our previous labeling experiments that were performed in a medium with about 30 µM LysoPC, we observed changes in the contribution of the different precursors to PC biosynthesis. The proportion of unlabeled PC decreased from 89.5% to 74.5%. The contributions of Cho-d_9_, Etn-d_4_, and Ser-d_3_ increased to 5.4, 14.5, and 3.2%, respectively. We also noted that in the serum-free medium, the amount of PC from Etn-d_4_ was higher than the one of PC from Cho-d_9_ (Figs. 2C and **4A**). PE and PS biosynthesis were not affected (Fig. 4A). In the absence of serum and when LysoPC was depleted, the contribution of the three labeled precursors to PC biosynthesis approximately doubled. The proportions of PC from Cho-d_9_ increased from 7.8% to 12.3% and of PC from Etn-d_4_ from 14.5% to 29.5%, (Fig. 4A). Under these conditions, 50.6% of PC was still found unlabeled, of which 20% was from the erythrocyte membrane. SM is the only other molecule containing a Cho motif that could possibly be used to synthesize PC. The addition of 20 µM SM to the serum-free medium, however, did not modify the contributions of the different pathways, showing that SM was not able to replace LysoPC for PC biosynthesis (Fig. 4A). In the absence of LysoPC, the remaining 30.6% of unlabeled PC were thus likely synthesized via the *Pf*PMT pathway from unlabeled Ser supplied by Hb degradation. In total, Ser contributed to 38.2% (7.6% from Ser-d_3_ and 30.6% from unlabeled Ser) of PC biosynthesis in the absence of LysoPC (Fig. 4B). To reveal the proportions of each precursor in the biosynthesis of PC, we recalculated the percentage excluding the contribution of host erythrocyte membrane (Fig. 4C). In the absence of any Cho source, *P. falciparum* parasites were apparently capable of compensating PC synthesis through the *Pf*PMT pathway. To confirm this assumption, we analyzed the growth behavior of WT 3D7 and of *pfpmtΔ* parasites in the presence or absence of different Cho sources. The WT strain was able to grow over a period of 5 days, even in the absence of serum, however, at a much reduced level in comparison to the standard culture medium containing 10% human serum (Fig. 4D). Complementing the serum-free medium with 20 µM Cho, the standard concentration in complete medium, almost doubled the growth of the parasites. This effect was further enhanced by a very high concentration of Cho (400 µM). Replacing 400 µM Cho by 20 µM LysoPC resulted in identical growth curves, indicating that the parasite uses LysoPC much more efficiently as a source of Cho. Finally, supplementation with 20 µM SM did not improve parasite growth, confirming that it cannot be used as a source for Cho. The *pfpmtΔ* strain showed a very similar behavior to the WT strain when 20 µM LysoPC or 400 µM Cho was present in the medium. However, these mutant parasites were totally unable to grow in the absence of Cho or when supplemented with either 20 µM Cho or SM. These results confirmed the importance of the *Pf*PMT pathway and the supply of Etn-derived metabolites to the PC-producing Kennedy pathway.

### Molecular species and source of PLs

Our lipidomic analyses revealed marked differences in the major molecular species of PC, PE, and PS between uRBCs and iRBCs (supplemental Tables S2–S4). For PC of the 3D7 strain, the main modifications were an increase in *P. falciparum*-iRBCs of PC 16:0–18:1 (from 22% to 32%) and of PC 16:0–16:0 (from 4% to 13%) and a decrease of PC 16:0–18:2 (from 23% to ∼14%) (supplemental Table S2). Concerning PE, the main change was a large increase of PE 16:0–18:2 from 8% to 26% (supplemental Table S3). Very drastic differences were observed for PS. In uRBCs, PS was predominantly composed of PS 18:0–20:4 (63%) but dropped to 32% in iRBCs, whereas the amount of PS 16:0–18:1 increased 12-fold (from 0.8% to ∼10%) (supplemental Table S4). No significant changes in the distribution of PL species were observed in iRBCs infected by the WT 3D7 strain compared with the *pfpmtΔ* strain. We analyzed in more detail the molecular species of each PL according to the precursor of their head group, focusing on molecular species that were present at more than 1% of total lipid. For PC in the 3D7 strain, two of the main molecular species, 16:0–18:0 and 16:0–18:1, varied when comparing unlabeled with the three labeled PCs (supplemental Table S5). These differences were not observed in the *pfpmtΔ* strain (supplemental Table S6) and were thus likely a consequence of unlabeled Ser as precursor, incorporated via the PMT pathway. This observation might suggest that PC can be synthesized from two different pools of DAG/FAs. For PE, we observed one significant difference for PE 16:0–18:2, one of the predominant species: this PE species from Etn was more abundant than the PE from Ser (PE from Ser-d_3_ or unlabeled PE) (supplemental Table S7). Finally, and in contrast to PC and PE, the profile of most of the molecular species of PS was very different between unlabeled PS (derived from Hb degradation) and Ser-d_3_-labeled PS (supplemental Tables S8 and S9), strongly suggesting the presence of two different pools of DAG/FAs and thus of two sites of PS synthesis in the parasite.

## DISCUSSION

Understanding the metabolism of *Plasmodium* is crucial to identifying new therapeutic targets in order to develop efficient novel antimalarial strategies. Although the *Plasmodium* PL metabolism has been studied (7, 37), it is not very well known how the multiple pathways interconnect and depend on each other. Our lipidomic analysis allowed us to quantify the incorporation of the three labeled precursors Cho, Etn, and Ser into the parasite’s main PLs (PC, PE, and PS). In this way, we gained unprecedented insight into the distribution of the labeled (and unlabeled) precursors in the PLs of *P. falciparum*-iRBCs.

We found that 89% of PC was synthesized from Cho via the CDP-Cho-dependent Kennedy pathway. The remaining 11% of PC was provided by the PMT pathway. We attributed the surprisingly high level of unlabeled PC to the use of LysoPC as Cho source. However, the parasite is capable of shifting PC synthesis to other sources (via the PMT pathway) if Cho and LysoPC are absent from the culture medium. There is thus a considerable level of flexibility in the metabolic pathways leading to PC synthesis. This might be of particular interest for the parasite as it passes through the very different environments during its complex life cycle, such as the blood meal in the mosquito gut, the mosquito hemolymph, and the human hepatocyte, in addition to the human RBC. Indeed, the importance of PC metabolism for sexual stage differentiation has been emphasized by the recent study of Brancucci et al. (26). They show that the enzymes of the PMT pathway are induced under LysoPC restriction conditions. These results corroborate our findings, showing that the parasite is able to use Etn and Ser to compensate for the absence of Cho (or LysoPC). At the liver stage, *Plasmodium* seems to rely on PC taken up from the hepatocyte host cell (38). However, KO of host CTα (CCT) did not affect parasite growth or merozoite formation, but impaired parasite survival, suggesting that *Plasmodium* is able to produce its own PC at this stage (38).

PE was synthesized about equally from Etn (43%) and from Ser (57%). Increasing the Etn-d_4_ concentration in the culture medium induced a sharp decrease of PE synthesized from Ser. The incorporation of Ser into PE is possible via two pathways: decarboxylation of PS or decarboxylation of the precursor Ser and synthesis via the CDP-Etn-dependent Kennedy pathway (Fig. 1). PSD activity has been demonstrated for *Plasmodium* (24), and a PSS-base-exchange enzyme (PSS2) is annotated in the genome database. Direct decarboxylation of Ser has been described in plants (23), and Ser decarboxylase catalytic activity has been suggested in *P. falciparum* (22). The presence of plant-like metabolic pathways in *Plasmodium* (such as the PMT enzyme) is not entirely surprising. Most apicomplexan parasites harbor a plastid-like organelle that originates from an ancestral endosymbiosis of a red alga (39). However, a gene coding for a Ser decarboxylase has not yet been identified in the *P. falciparum* genome. We could demonstrate the presence of Etn-d_3_ (Etn derived from Ser-d_3_) in the parasite, but this cannot serve as a proof for Ser decarboxylase activity, because Etn-d_3_ can also be obtained through three enzymatic steps: *1*) incorporation of Ser-d_3_ into PS-d_3_ by the PSS2; *2*) decarboxylation of PS-d_3_ to PE-d_3_ by the PSD; and, finally, *3*) Etn-d_3_ liberation via a base exchange process of the PSS2 (Fig. 1). The existence of Ser decarboxylase activity in the parasite could be demonstrated in labeling experiments using a *pfpsd* KO strain if this gene is not essential: the presence of Ser-d_3_ labeling in the metabolites of the PE Kennedy pathway would then depend on Ser decarboxylase activity.

While the CDP-Cho- and CDP-Etn-dependent pathways are the major pathways, respectively, for PC and PE synthesis in mammalian cells, acylation of LysoPC or of LysoPE by the Land’s cycle (40) could contribute to PL synthesis and has been shown to be active in mature RBCs (41, 42). So far, there is no indication that *Plasmodium* incorporates LysoPC directly into PC via acylation. In our experiments, if LysoPC acylation was significantly present, the percentage of unlabeled molecules (derived from LysoPC) would be expected to be higher for PC than for the reaction intermediates of the Kennedy pathway. However, we observed an equally high percentage of about 90% of unlabeled molecules for all reaction substrates, i.e., Cho, P-Cho, and CDP-Cho, and for the lipid PC. In addition, Brancucci et al. (26), using ^2^H-choline-labeled LysoPC, showed that ∼70% of free Cho of the parasite was labeled. All these data therefore indicate that LysoPC is not integrated as such into PC through acylation, but is first degraded to Cho. LysoPC could be cleaved first by a lysophospholipase to give glycero-3-P-Cho and then by the glycerophosphodiester phosphodiesterase (GDPD) to produce Cho. The presence of lysophospholipases in the RBCs and of the GDPD in the parasitophorous vacuole (43) suggests that the cleavage of LysoPC occurs before entering the parasite. This is consistent with the antimalarial activity described for Cho analogs (15, 44). Indeed, these potent antimalarials, of which T3/albitiazolium is the lead compound, are shown to inhibit the Cho uptake into the parasite and PC biosynthesis (15).

Regarding LysoPE, the concentration provided by the serum is very low (<1 µM) (45, 46), and its acylation could thus only be of marginal importance for PE synthesis.

The only route to synthesize PS is by PSS2. Using up to 1 mM labeled Ser in the culture medium in our experiments revealed that the parasite preferentially (>80%) incorporated unlabeled Ser into PS and also into PE and PC. Since Ser biosynthesis from 3-phosphoglycerate, as observed in mammalian cells, is absent in *Plasmodium*, the only possible internal source of Ser is proteolysis of Hb (47). This would make the parasite self-sufficient for this precursor, which likely is advantageous for its survival at the blood stage. We attempted to evaluate the contribution of Hb-derived Ser, by performing our parasite-labeling assays in the presence of the cysteine proteases inhibitor E-64 (10 µM) to temporarily reduce Hb degradation (data not shown). The proportion of unlabeled PS and PE decreased by 5–7%, confirming that Hb-derived Ser contributed to the pool used for PL synthesis. However, the toxic effect of E-64 treatments over longer periods precluded a more detailed analysis. Another way to directly demonstrate that Ser is provided by Hb would be to perform experiments with uRBCs that contain Hb labeled during erythropoiesis.

In conclusion, our lipidomic approach allowed us to gain a global view of the PL metabolism and to establish a new PL metabolism map revealing the relative contributions of the different pathways. *P. falciparum* possesses a multiplicity of metabolic routes and is able, to some extent, to adapt to variations in the availability of PL precursors. Given the interconnections of the different pathways and their compensation, an effective pharmacological strategy should focus on enzymes that are essential or that are bottlenecks in these pathways. The CTP:P-Cho cytidylyltransferase and the Cho/Etn-phosphotransferase (CEPT) would be such candidates for PC synthesis, whereas our results indicate that Cho kinase activity might be dispensable at least in the presence of high concentrations of Etn and Ser. It is of note that in *P. falciparum*, a single enzyme (CEPT) catalyzes the formation of PC and PE from CDP-Cho and CDP-Etn, respectively (7). This enzyme has been shown to be essential for parasite survival in a rodent malaria model (48). Another interesting target could be PSS2, the unique enzyme to provide PS.

## Supplementary Material

Supplemental Data
