# Supplementary material for: Contribution of the precursors and interplay of the pathways in the phospholipid metabolism of the malaria parasite
Source: J Lipid Res. 2018 May 31;59(8):1461–71. doi: 10.1194/jlr.M085589 (PMC6071779; doi:10.1194/jlr.M085589)
Supplement: Supplemental Data [file supp_59_8_1461__index.html]

Contribution of the precursors and interplay of the pathways in the phospholipid metabolism of the malaria parasite — Contribution of the precursors and interplay of the pathways in the phospholipid metabolism of the malaria parasite — Supplemental Data 

# Contribution of the precursors and interplay of the pathways in the phospholipid metabolism of the malaria parasite

## Supplemental Data

- Supplemental information (.pdf, 664 KB) - Supplemental tables and figures
