# Supplementary material for: Contribution of the precursors and interplay of the pathways in the phospholipid metabolism of the malaria parasite
Source: J Lipid Res. 2018 May 31;59(8):1461–71. doi: 10.1194/jlr.M085589 (PMC6071779; doi:10.1194/jlr.M085589)
Supplement: Supplemental Data [file 10.1194_M085589_jlr.M085589-1.pdf]

SUPPLEMENTAL INFORMATION

Contribution of the precursors and interplay of the pathways in the phospholipid metabolism of the malaria parasite

Sharon Wein[1*#], Salma Ghezal[2#], Corinne Buré[3], Marjorie Maynadier[1], Christian Périgaud[2], Henri J. Vial[1], Isabelle Lefebvre-Tournier[2], Kai Wengelnik[1], Rachel Cerdan[1]

[1]Dynamique des Interactions Membranaires Normales et Pathologiques, UMR 5235, CNRS-Université de Montpellier, cc 107, place Eugène Bataillon, 34095 Montpellier Cedex 05, France.

[2]Institut des Biomolécules Max Mousseron, UMR 5247, CNRS- Université de Montpellier, cc 1705, place Eugène Bataillon, 34095 Montpellier Cedex 05, France.

[3]Chimie et Biologie des Membranes et des Nanoobjets, UMR 5248, Centre de Génomique Fonctionnelle, BP 68, Université Bordeaux 2, 146, rue Léo Saignat, 33076 Bordeaux cedex, France.

[#] These authors contributed equally to this work.

Supplemental Table S1. Labeling of PC, PE, PS, their precursors and the reaction intermediates found in *P. falciparum*-iRBC.

	Metabolite labeling (%)			
	**UL**	**d_3**	**d_4**	**d_9**
**PC**	89.5 ± 3.0	0.6 ± 0.3	4.5 ± 1.4	5.4 ± 2.1
**PE**	56.8 ± 12.0	8.5 ± 1.0	34.7 ± 11.0	NA
**PS**	86 ± 3.4	14.0 ± 3.4	NA	NA
**Cho**	92.4 ± 2.3	NA	NA	7.6 ± 2.3
**P-Cho**	89.2 ± 1.3	0.8 ± 0.5	4.6 ± 1.8	5.5 ± 1.5
**CDP-Cho**	87.7 ± 7.7	0.7 ± 0.7	6.0 ± 0.3	6.9 ± 6.0
**P-Etn**	42.7 ± 15.5	6.7 ± 4.6	50.7 ± 15.2	NA
**CDP-Etn**	36.8 ± 23.0	6.3 ±2.0	56.8 ± 21.0	NA
**Ser**	71.9 ± 17.8	28.1 ± 17.8	NA	NA

Cells were cultured in presence of human serum and 20 µM Cho-d_9, 10 µM Etn-d_4 and 140 µM Ser-d_3.Values are the mean of at least 3 independent experiments ± s.d.

UL: unlabeled; d_9: d_9 labeling (from choline-d_9), d_4 labeling (from ethanolamine-d_4), d_3 labeling (from serine-d_3), NA: not applicable.

Supplemental Table S2. Phosphatidylcholine species in uRBC and *P. falciparum*-iRBC (3D7 and *pfpmtΔ* strains). *P. falciparum*-iRBC were incubated 48h in culture medium supplemented with 10% human serum in the presence of 20 µM Cho-d_9, 10 µM Etn-d_4 and 140 µM Ser-d_3. After incubation, PLs were extracted, identified and quantified by LC-MS/MS. Gray color indicate the main changes in PC molecular species between RBCs and iRBCs.

	**RBC (% of total PC)**		**3D7 *P. f.*-iRBC (% of total PC)**		***pfpmtΔ P. f.*-iRBC (% of total PC)**	
	Mean	**s.e.m. (n=3)**	**Mean**	**s.e.m. (n=7)**	Mean	**s.e.m. (n=3)**
**PC 14:0-16:1**	**3.67**	0.56	**0.92**	0.16	**0.66**	0.11
**PC 16:0/16:0**	**4.28**	0.11	**13.01**	1.29	**15.39**	1.27
**PC 14:0-18:1**	**1.11**	0.24	**1.96**	0.48	**1.25**	0.05
**PC 14:0-18:2**	**0.16**	0.01	**0.28**	0.05	**0.17**	0.01
**PC 16:0-18:0**	**5.78**	2.31	**11.70**	3.22	**9.12**	1.40
**PC 16:0-18:1**	**22.57**	1.21	**32.42**	1.94	**29.60**	0.81
**PC 16:0-18:2**	**22.91**	0.49	**14.99**	0.89	**13.95**	0.79
**PC 18:0-18:1**	**5.46**	0.55	**6.12**	0.33	**6.52**	0.54
**PC 18:0-18:2 + PC 18:1/18:1**	**10.75**	1.02	**5.89**	0.33	**5.71**	0.39
**PC 16:0-20:3 + PC 18:1-18:2**	**6.46**	0.37	**3.21**	0.19	**3.71**	0.36
**PC 16:1-20:3 + PC 16:0-20:4 + PC 18:2/18:2**	**7.41**	0.73	**4.28**	0.30	**6.73**	1.12
**PC 38:3**	**1.17**	0.16	**0.69**	0.06	**0.97**	0.01
**PC 18:0-20:4 + PC 18:1-20:3**	**3.69**	0.08	**1.73**	0.15	**2.51**	0.23
**PC 38:5**	**1.91**	0.27	**1.15**	0.10	**1.61**	0.15
**PC 16:0-22:6**	**2.15**	0.24	**1.30**	0.10	**1.78**	0.19
**PC 40:4**	**0.15**	0.01	**0.07**	0.01	**0.10**	0.01
**PC 40:5**	**0.34**	0.07	**0.17**	0.02	**0.24**	0.01

PC: phosphatidylcholine

Supplemental Table S3. Phosphatidylethanolamine species in uninfected RBC and *P. falciparum*-iRBC (3D7 and *pfpmtΔ* strains). *P. falciparum*-iRBC were incubated 48h in culture medium supplemented with 10% human serum in the presence of 20 µM Cho-d_9, 10 µM Etn-d_4 and 140 µM Ser-d_3. After incubation, PLs were extracted, identified and quantified by LC-MS/MS. Gray color indicate the main change in PE molecular species between RBCs and iRBCs.

	**RBC (% of total PE)**		**3D7 *P. f.*-iRBC (% of total PE)**		***pfpmtΔ P. f.*-iRBC (% of total PE)**	
**Species**	**Mean**	**s.e.m. (n=3)**	**Mean**	**s.e.m. (n=3)**	**Mean**	**s.e.m. (n=3)**
**PE 14:0/18:1**	**0.22**	0.03	**0.55**	0.12	**0.47**	0.05
**PE 14:0/18:2**	**0.09**	0.01	**0.50**	0.14	**0.47**	0.08
**PE 16:0/16:0**	**0.77**	0.12	**2.39**	0.48	**2.24**	0.83
**PE 16:0/18:0**	**0.24**	0.01	**0.43**	0.14	**1.05**	0.34
**PE 16:0/18:1**	**23.49**	0.68	**29.14**	1.04	**27.46**	0.80
**PE 16:0/18:2**	**8.17**	0.38	**26.90**	0.91	**24.60**	1.49
**PE 16:0/20:1**	**0.23**	0.01	**0.14**	0.06	**0.21**	0.03
**PE 16:0/20:3**	**0.39**	0.20	**0.42**	0.12	**0.00**	0.00
**PE 16:0/20:4**	**12.54**	1.16	**9.92**	0.90	**13.35**	0.83
**PE 16:0/22:4**	**3.84**	0.12	**0.83**	0.09	**0.64**	0.06
**PE 16:0/22:5**	**2.24**	0.21	**0.69**	0.16	**1.17**	0.02
**PE 16:0/22:6**	**2.58**	0.39	**1.12**	0.12	**1.13**	0.06
**PE 18:0/18:1**	**6.85**	0.27	**4.82**	0.64	**5.88**	0.40
**PE 18:0/18:2**	**2.25**	0.15	**3.32**	0.25	**4.89**	0.43
**PE 18:0/20:3**	**0.59**	0.30	**0.78**	0.18	**1.24**	0.27
**PE 18:0/20:4**	**9.02**	0.38	**4.11**	0.38	**4.72**	0.71
**PE 18:0/22:4**	**1.30**	0.09	**0.27**	0.07	**0.24**	0.01
**PE 18:1/18:1**	**7.57**	0.55	**3.23**	0.46	**2.87**	0.34
**PE 18:1/18:2**	**7.30**	0.38	**2.92**	0.68	**4.06**	0.26
**PE 18:1/20:3**	**0.93**	0.07	**0.12**	0.06	**0.00**	0.00
**PE 18:1/20:4**	**7.66**	0.40	**1.60**	0.12	**1.61**	0.11
**PE 18:1/22:4**	**1.08**	0.09	**0.10**	0.04	**0.12**	0.01
**PE 18:2/18:2**	**0.66**	0.02	**0.61**	0.30	**1.57**	0.13

PE: phosphatidylethanolamine

Supplemental Table S4. Phosphatidylserine species in uninfected RBC and *P. falciparum*-iRBC (3D7 and *pfpmtΔ* strains). *P. falciparum*-iRBC were incubated 48h in culture medium supplemented with 10% human serum in the presence of 20 µM Cho-d_9, 10 µM Etn-d_4 and 140 µM Ser-d_3. After incubation, PLs were extracted, identified and quantified by LC-MS/MS. Gray color indicate the main changes in PS molecular species between RBCs and iRBCs.

	**RBC (% of total PS)**		**3D7 *P. f.*-iRBC (% of total PS)**		***pfpmtΔ P. f.*-iRBC (% of total PS)**	
**Species**	**Mean**	**s.e.m. (n=3)**	**Mean**	**s.e.m. (n=7)**	**Mean**	**s.e.m. (n=3)**
**PS 16:0-18:0**	**3.85**	0.77	**3.54**	0.63	**4.25**	0.53
**PS 16:0-18:1**	**0.81**	0.16	**10.62**	1.41	**12.85**	1.42
**PS 16:0-18:2**	**0.00**	0.00	**4.56**	0.89	**5.90**	0.94
**PS 16:0-20:1**	**0.00**	0.00	**0.19**	0.16	**0.00**	0.00
**PS 16:0-22:4**	**0.15**	0.10	**0.00**	0.00	**0.00**	0.00
**PS 16:0-22:5**	**0.00**	0.00	**0.03**	0.03	**0.00**	0.00
**PS 16:0-22:6**	**0.00**	0.00	**0.39**	0.30	**0.00**	0.00
**PS 18:0-18:1**	**9.44**	0.69	**14.96**	0.93	**16.36**	1.46
**PS 18:0-18:2**	**2.85**	0.12	**9.26**	0.51	**11.28**	0.20
**PS 18:0-20:3**	**6.60**	0.37	**4.83**	0.69	**5.33**	0.26
**PS 18:0-20:4**	**63.11**	1.97	**32.72**	2.45	**29.04**	1.61
**PS 18:0-22:4**	**8.58**	0.76	**4.66**	0.86	**3.17**	0.39
**PS 18:1/18:1**	**0.79**	0.07	**5.83**	1.02	**3.91**	0.15
**PS 18:1-18:2**	**0.16**	0.03	**3.41**	0.63	**3.47**	0.12
**PS 18:1-20:3**	**0.40**	0.11	**0.50**	0.19	**0.00**	0.00
**PS 18:1-20:4**	**3.86**	1.02	**3.80**	0.39	**4.44**	0.43
**PS 18:1-22:4**	**0.29**	0.15	**0.00**	0.00	**0.00**	0.00
**PS 18:2/18:2**	**0.00**	0.00	**0.24**	0.12	**0.00**	0.00

PS: phosphatidylserine

Supplemental Table S5. Molecular species of PC in the 3D7 strain according to the precursor of PLs. *P. falciparum*-iRBC were incubated 48h in culture medium supplemented with 10% human serum in the presence of 20 µM Cho-d_9, 10 µM Etn-d_4 and 140 µM Ser-d_3. After incubation, PLs were extracted, identified and quantified by LC-MS/MS. Values are the mean ± s.e.m. (n=4). Gray color indicate the main changes in PC molecular species between unlabeled and labeled PC.

	**Unlabeled PC**		**PC-d_3**		**PC-d_4**		**PC-d_9**	
	**mean (%)**	**s.e.m (%)**	**mean (%)**	**s.e.m (%)**	**mean (%)**	**s.e.m (%)**	**mean (%)**	**s.e.m (%)**
**PC 14:0-16:1**	**0.64**	0.19	**0**	0	**0.06**	0.04	**0.05**	0.03
**PC 16:0/16:0**	**12.05**	1.46	**13.01**	0.99	**12.78**	1.72	**12.79**	1.83
**PC 14:0-18:1**	**2.26**	0.67	**1.15**	0.39	**2.53**	0.62	**2.57**	0.53
**PC 14:0-18:2**	**0.32**	0.07	**0.07**	0.07	**0.24**	0.08	**0.26**	0.09
**PC 16:0-18:0**	**13.04**	3.29	**4.87**	2.14	**3.70**	0.98	**3.63**	0.82
**PC 16:0-18:1**	**32.67**	3.07	**43.45**	5.35	**40.22**	1.22	**40.63**	1.63
**PC 16:0-18:2**	**14.76**	1.12	**17.59**	2.21	**16.76**	1.36	**15.83**	0.89
**PC 18:0-18:1**	**5.87**	0.29	**5.89**	0.41	**5.81**	0.71	**6.31**	0.32
**PC 18:0-18:2 + PC 18:1/18:1**	**5.82**	0.54	**4.91**	1.86	**5.76**	0.16	**5.82**	0.21
**PC 16:0-20:3 + PC 18:1-18:2**	**3.27**	0.43	**2.58**	0.89	**3.29**	0.16	**3.43**	0.16
**PC 16:1-20:3 + PC 16:0-20:4 + PC 18:2/18:2**	**4.15**	0.44	**3.04**	1.25	**4.51**	0.18	**4.36**	0.13
**PC 36:8**	**0.18**	0.00	**0**	0	**0.19**	0.00	**0.20**	0.00
**PC 38:3**	**0.65**	0.08	**0.14**	0.14	**0.40**	0.15	**0.38**	0.13
**PC 18:0-20:4 + PC 18:1-20:3**	**1.66**	0.29	**1.31**	0.58	**1.39**	0.18	**1.23**	0.42
**PC 38:5**	**1.23**	0.23	**0.56**	0.42	**0.92**	0.33	**1.00**	0.35
**PC 16:0-22:6**	**1.35**	0.22	**0.84**	0.50	**1.38**	0.26	**1.44**	0.14
**PC 40:4**	**0.06**	0.02	**0**	0	**0.05**	0.02	**0.06**	0.03
**PC 40:5**	**0.16**	0.04	**0**	0	**0.14**	0.06	**0.17**	0.06
Total	100%		100%		100%		100%	

Supplemental Table S6. Molecular species of PC in *pfpmtΔ* strain according to the source of PLs. *P. falciparum*-iRBC were incubated 48h in culture medium supplemented with 10% human serum in the presence of 20 µM Cho-d_9, 10 µM Etn-d_4 and 140 µM Ser-d_3. After incubation, phospholipids were extracted, identified and quantified by LC-MS/MS. Results are mean ± s.e.m. (n=3).

	**Unlabeled PC**		**PC-d_9**	
	**mean (%)**	**s.e.m (%)**	**mean (%)**	**s.e.m (%)**
**PC 14:0-16:1**	**0.67**	0.11	**0.00**	0.00
**PC 16:0/16:0**	**15.47**	1.27	**11.15**	1.53
**PC 14:0-18:1**	**1.24**	0.04	**1.96**	0.75
**PC 14:0-18:2**	**0.17**	0.01	**0.00**	0.00
**PC 16:0-18:0**	**9.16**	1.42	**7.54**	0.99
**PC 16:0-18:1**	**29.62**	0.77	**28.18**	3.90
**PC 16:0-18:2**	**13.95**	0.79	**13.87**	0.79
**PC 18:0-18:1**	**6.54**	0.54	**5.66**	0.73
**PC 18:0-18:2 + PC 18:1/18:1**	**5.69**	0.41	**6.79**	0.58
**PC 16:0-20:3 + PC 18:1-18:2**	**3.69**	0.37	**4.58**	0.36
**PC 16:1-20:3 + PC 16:0-20:4 + PC 18:2/18:2**	**6.68**	1.12	**9.24**	1.36
**PC 38:3**	**0.93**	0.03	**2.84**	0.73
**PC 18:0-20:4 + PC 18:1-20:3**	**2.51**	0.23	**2.58**	0.12
**PC 38:5**	**1.58**	0.16	**2.83**	0.45
**PC 16:0-22:6**	**1.76**	0.20	**2.55**	0.09
**PC 40:4**	**0.11**	0.01	**0.00**	0.00
**PC 40:5**	**0.24**	0.01	**0.22**	0.22
Total	100%		100%	

Supplemental Table S7. Molecular species of PE in 3D7 strain according to the source of PLs. *P. falciparum*-iRBC were incubated 48h in culture medium supplemented with 10% human serum in the presence of 20 µM Cho-d_9, 10 µM Etn-d_4 and 140 µM Ser-d_3. After incubation, phospholipids were extracted, identified and quantified by LC-MS/MS. Results are mean ± s.e.m. (n=4). Gray color indicate the main change in PE molecular species between unlabeled and labeled PE.

	**Unlabeled PE**		**PE-d_3**		**PE-d_4**	
	**mean (%)**	**s.e.m (%)**	**mean (%)**	**s.e.m (%)**	**mean (%)**	**s.e.m (%)**
**PE 14:0-18:1**	**0.64**	0.10	**0.47**	0.24	**0.60**	0.17
**PE 14:0-18:2**	**0.48**	0.09	**0.50**	0.32	**0.51**	0.21
**PE 16:0/16:0**	**1.97**	0.52	**2.17**	0.61	**2.03**	0.54
**PE 16:0-18:0**	**0.59**	0.19	**0.07**	0.07	**0.48**	0.14
**PE 16:0-18:1**	**30.25**	0.94	**31.01**	2.12	**31.80**	1.51
**PE 16:0-18:2**	**24.51**	1.47	**24.00**	1.41	**31.43**	1.98
**PE 16:0-20:1**	**0.18**	0.08	**0.05**	0.05	**0.12**	0.07
**PE 16:0-20:3**	**0.55**	0.19	**0.73**	0.73	**0.40**	0.23
**PE 16:0-20:4**	**9.38**	0.79	**10.55**	2.02	**9.50**	1.09
**PE 16:0-22:4**	**1.30**	0.08	**0.43**	0.16	**0.77**	0.20
**PE 16:0-22:5**	**0.76**	0.27	**0.25**	0.14	**0.44**	0.17
**PE 16:0-22:6**	**1.25**	0.16	**0.76**	0.57	**1.19**	0.35
**PE 18:0-18:1**	**6.57**	0.81	**7.26**	0.16	**4.57**	0.41
**PE 18:0-18:2**	**4.01**	0.64	**4.24**	1.03	**2.92**	0.53
**PE 18:0-20:3**	**0.91**	0.09	**0.22**	0.13	**0.84**	0.30
**PE 18:0-20:4**	**4.97**	0.33	**4.56**	2.53	**3.00**	0.48
**PE 18:0-22:4**	**0.53**	0.05	**0.05**	0.05	**0.19**	0.04
**PE 18:1/18:1**	**4.83**	1.01	**5.86**	1.24	**2.52**	0.40
**PE 18:1-18:2**	**3.18**	0.14	**4.32**	0.31	**4.13**	2.57
**PE 18:1-20:3**	**0.33**	0.11	**0.08**	0.05	**0.20**	0.11
**PE 18:1-20:4**	**2.07**	0.16	**1.73**	0.32	**1.03**	0.25
**PE 18:1-22:4**	**0.25**	0.05	**0.01**	0.01	**0.00**	0.00
**PE 18:2/18:2**	**0.52**	0.05	**0.71**	0.29	**1.35**	1.22
Total	100%		100%		100%	

Supplemental Table S8. Molecular species of PS in 3D7 strain according to the source of PLs. *P. falciparum*-iRBC were incubated 48h in culture medium supplemented with 10% human serum in the presence of 20 µM Cho-d_9, 10 µM Etn-d_4 and 140 µM Ser-d_3. After incubation, phospholipids were extracted, identified and quantified by LC-MS/MS. Results are mean ± s.e.m. (n=4). Significant differences are shown ns: not significant, * p<0.05, ** p<0.01, *** p<0.001, **** p<0.0001.

	**Unlabeled PS**		**PS-d_3**		**Significant difference**
	**mean (%)**	**s.e.m (%)**	**mean (%)**	**s.e.m (%)**	
**PS 16:0-18:0**	**2.91**	0.75	**0.00**	0.00	**
**PS 16:0-18:1**	**11.23**	0.92	**20.45**	0.99	**
**PS 16:0-18:2**	**3.74**	0.18	**5.28**	1.21	ns
**PS 16:0-20:1**	**1.95**	0.85	**1.29**	0.71	ns
**PS 18:0-18:1**	**15.58**	0.68	**20.92**	3.89	ns
**PS 18:0-18:2**	**8.65**	0.30	**15.11**	0.20	****
**PS 18:0-20:3**	**4.50**	0.26	**0.11**	0.11	****
**PS 18:0-20:4**	**31.79**	0.76	**10.80**	5.40	**
**PS 18:0-22:4**	**4.13**	0.65	**0.00**	0.00	**
**PS 18:1/18:1**	**6.98**	1.46	**14.05**	1.18	*
**PS 18:1-18:2**	**3.24**	0.20	**7.21**	1.11	**
**PS 18:1-20:3**	**0.90**	0.18	**0.00**	0.00	**
**PS 18:1-20:4**	**3.74**	0.41	**4.78**	1.01	ns
Total	100%		100%		

Supplemental Table 9. Molecular species of PS in *pfpmtΔ* strain according to the source of PLs. *P. falciparum*-iRBC were incubated 48h in culture medium supplemented with 10% human serum in the presence of 20 µM Cho-d_9, 10 µM Etn-d_4 and 140 µM Ser-d_3. After incubation, phospholipids were extracted, identified and quantified by LC-MS/MS. Results are mean ± s.e.m. (n=3).

	**PS-d_0**		**PS-d_3**		**Significant difference**
	**mean (%)**	**s.e.m (%)**	**mean (%)**	**s.e.m (%)**	
**PS 16:0-18:0**	**4.91**	0.65	**0.00**	0.00	**
**PS 16:0-18:1**	**12.04**	1.52	**18.14**	0.58	*
**PS 16:0-18:2**	**5.57**	0.77	**7.82**	1.89	ns
**PS 18:0-18:1**	**15.88**	1.49	**19.49**	1.53	ns
**PS 18:0-18:2**	**11.25**	0.09	**11.62**	0.90	ns
**PS 18:0-20:3**	**5.55**	0.15	**3.98**	1.01	ns
**PS 18:0-20:4**	**30.26**	1.65	**21.09**	1.06	**
**PS 18:0-22:4**	**3.65**	0.41	**0.00**	0.00	***
**PS 18:1/18:1**	**3.44**	0.21	**6.94**	0.08	****
**PS 18:1-18:2**	**3.29**	0.11	**4.64**	0.26	**
**PS 18:1-20:4**	**4.16**	0.40	**6.28**	0.59	*
Total	100%		100%		

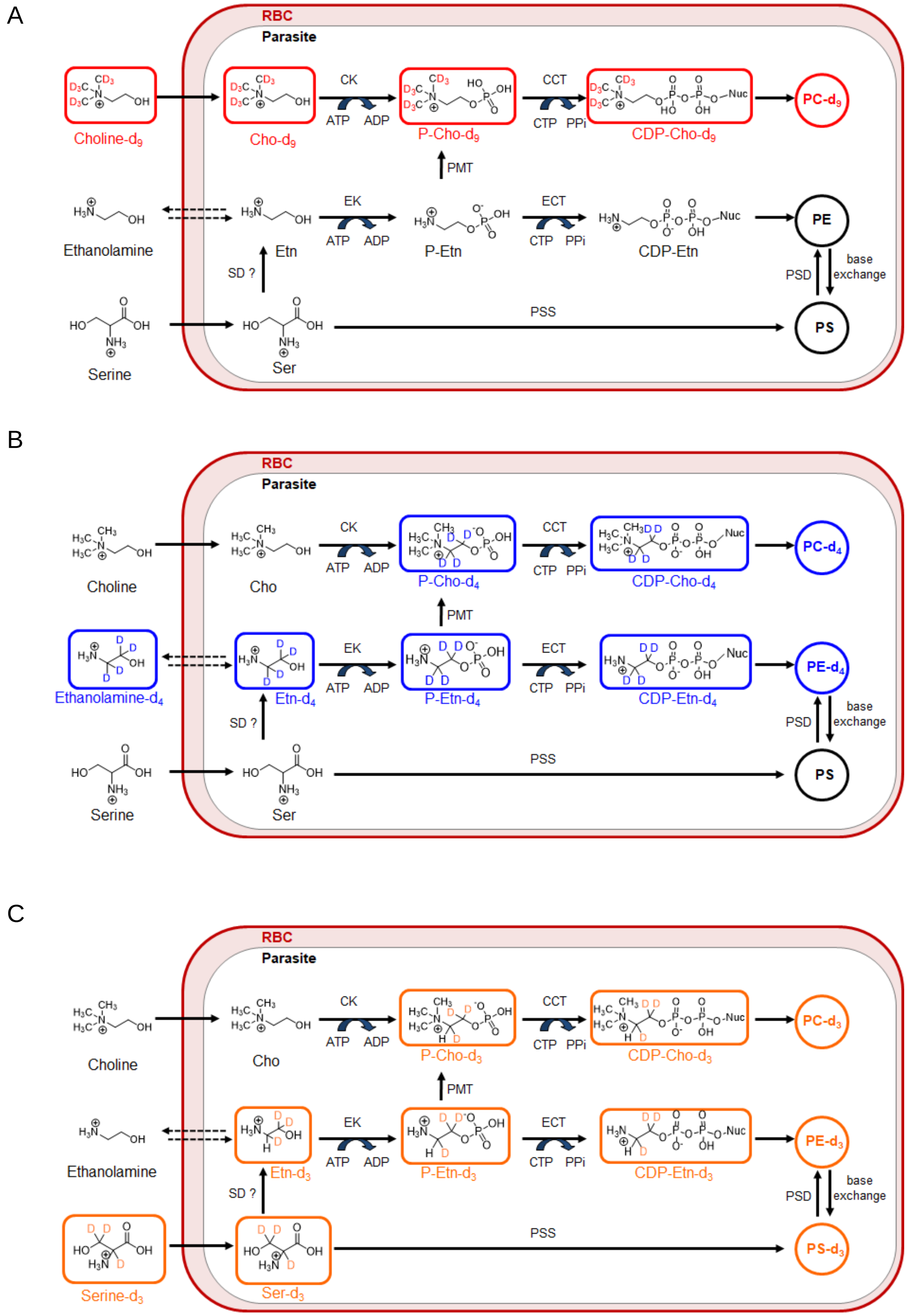

Supplemental Figure S1: Principle of deuterium labeling of *P. falciparum* PL metabolism.
P. falciparum-iRBCs were incubated 48 h or 96 h in several culture media in the presence of 20 µM Cho-d_9, 10 µM Etn-d_4 and 140 µM Ser-d_3. After incubation, PL and reaction intermediates were extracted and quantified by LC-MS/MS. **(A)** Molecules labeled with “d_9” were synthesized from Cho-d_9. **(B)** Molecules labeled with “d_4” were synthesized from Etn-d_4. **(C)** Molecules labeled with “d_3” were synthesized from Ser-d_3. The contributions of Cho-d_9, Etn-d_4 and Ser-d_3 to biosynthesis of PLs can thus be determined.

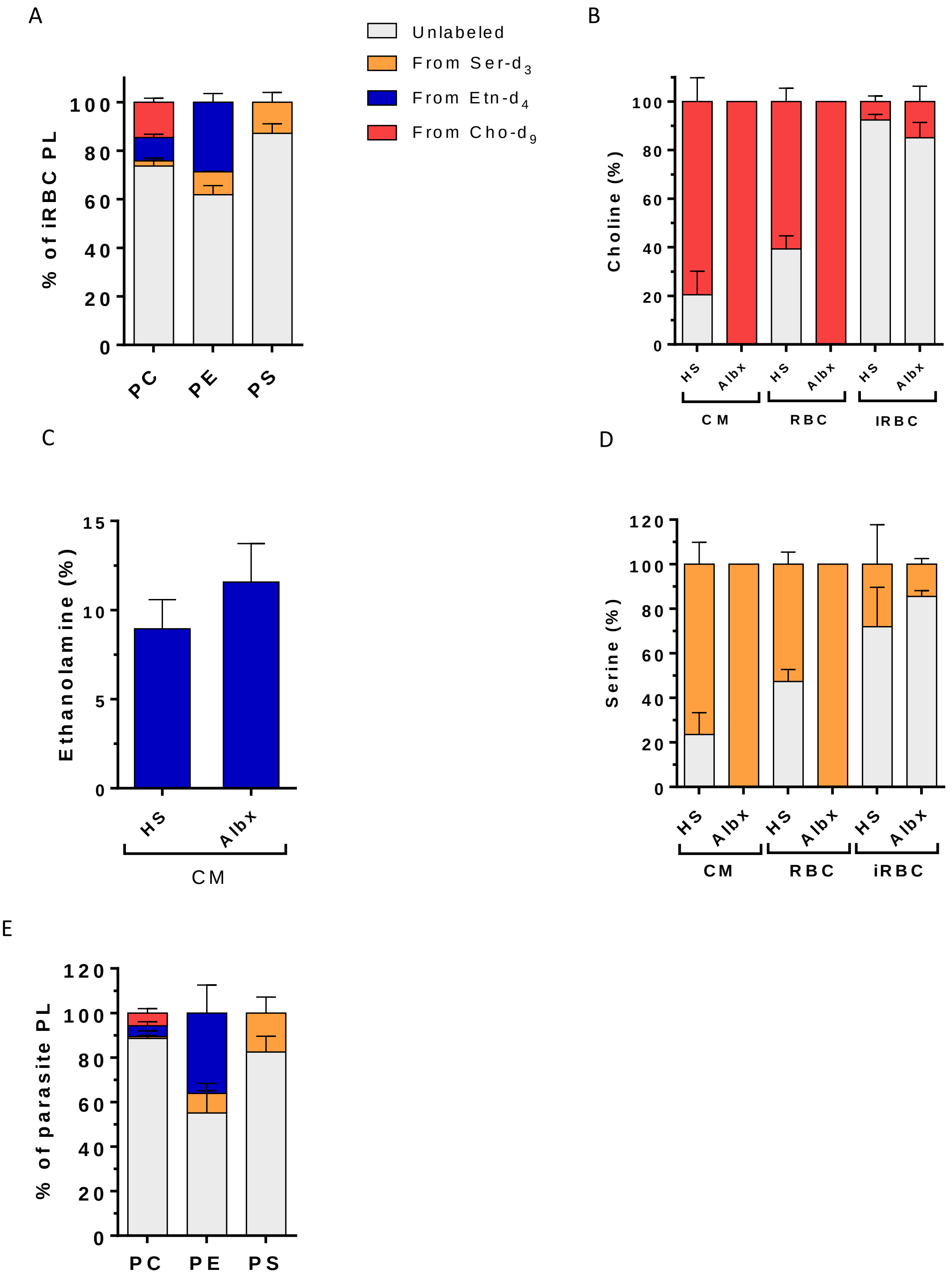

Supplemental Figure S2: Quantification and origin of PC, PE and PS in *P. falciparum*-iRBC and isolated parasites. *P. falciparum*-iRBC were incubated 48h in culture medium supplemented with 0.5% albumax I in the presence of 20 µM Cho-d_9, 10 µM Etn-d_4 and 140 µM Ser-d_3. After incubation, a fraction was treated with saponin to obtain isolated parasites. After extraction, phospholipids and reaction intermediates were quantified by LC-MS/MS. **(A)** Quantification of total PC, PE and PS in iRBC. **(B to D)** Quantification of the precursors Cho (B), Etn (C) and serine (D) in culture medium (CM) and RBC in the presence of 10% human serum (HS) or 0.5% albumax (Albx) **(E)** Quantification of total PC, PE and PS in isolated parasites. The same color code is used for all the panels.

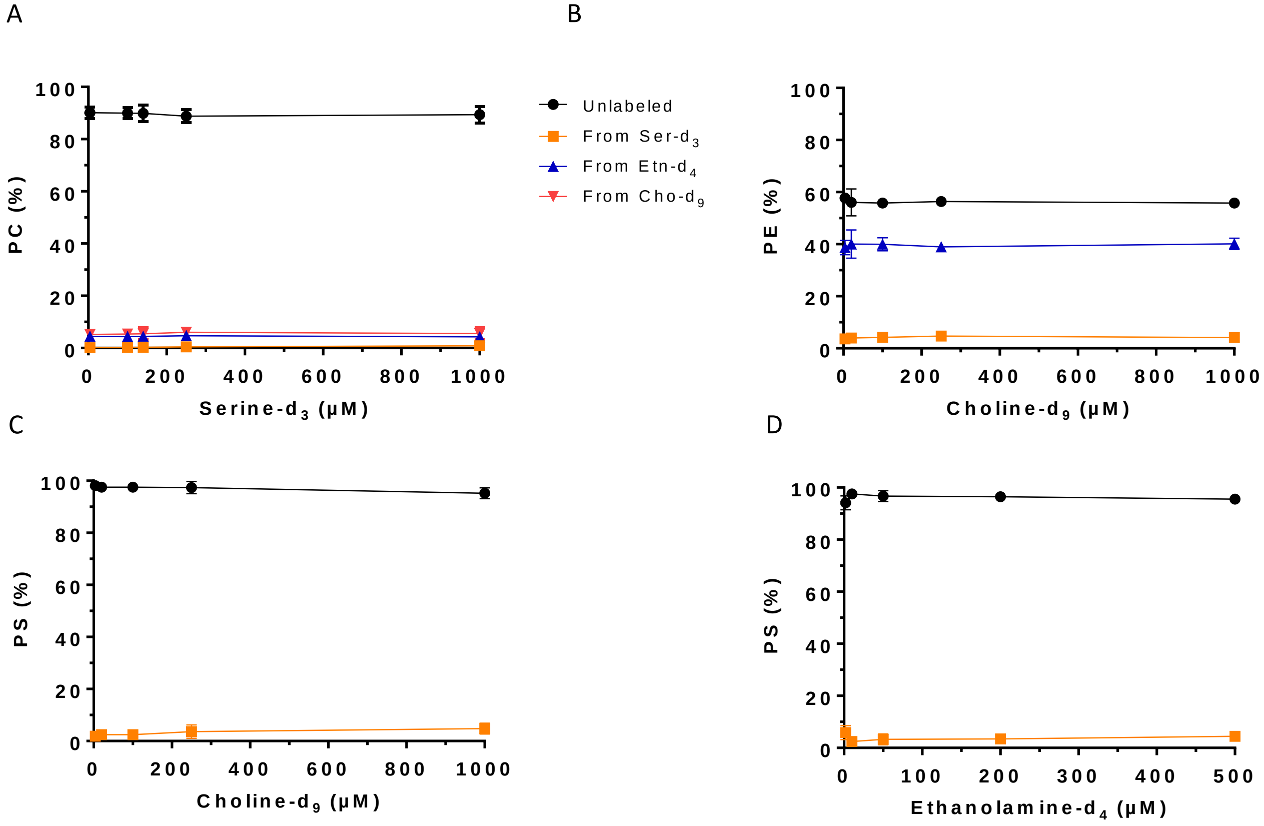

Supplemental Figure S3: Effect of varying precursor concentrations on the PC, PE and PS biosynthesis pathways in *P. falciparum*-iRBC. *P. falciparum*-iRBCs were incubated 96 h in culture medium supplemented with 10% human serum in the presence of increasing concentrations of Cho-d_9, Etn-d_4 or Ser-d_3 while the two others labeled precursors remained at fixed concentrations. Fixed concentrations were 20 µM Cho-d_9, 10 µM Etn-d_4 and 140 µM Ser-d_3. After incubation, PLs were quantified by LC-MS/MS. **(A)** Impact of Ser-d_3 concentration on PC biosynthesis. **(B)** Impact of Cho-d_9 concentration on PE biosynthesis. **(C)** Impact of Cho-d_9 and Etn-d_4 **(D)** concentrations on PS biosynthesis. Results are shown as means ± s.d. of three independant experiments. The same color code was used for all the panels.
